# Advances in the oral microbiota and rapid detection of oral infectious diseases

**DOI:** 10.3389/fmicb.2023.1121737

**Published:** 2023-02-06

**Authors:** Xuan Xiao, Shangfeng Liu, Hua Deng, Yuhan Song, Liang Zhang, Zhifeng Song

**Affiliations:** ^1^Department of Oral Mucosa, Shanghai Stomatological Hospital, Fudan University, Shanghai, China; ^2^Shanghai Key Laboratory of Craniomaxillofacial Development and Diseases, Shanghai Stomatological Hospital, Fudan University, Shanghai, China; ^3^Translational Medicine Center, Guangdong Women and Children Hospital, Guangzhou, China

**Keywords:** oral cavity, microbiota, infectious diseases, rapid diagnosis, precise treatment

## Abstract

Several studies have shown that the dysregulation of the oral microbiota plays a crucial role in human health conditions, such as dental caries, periodontal disease, oral cancer, other oral infectious diseases, cardiovascular diseases, diabetes, bacteremia, and low birth weight. The use of traditional detection methods in conjunction with rapidly advancing molecular techniques in the diagnosis of harmful oral microorganisms has expanded our understanding of the diversity, location, and function of the microbiota associated with health and disease. This review aimed to highlight the latest knowledge in this field, including microbial colonization; the most modern detection methods; and interactions in disease progression. The next decade may achieve the rapid diagnosis and precise treatment of harmful oral microorganisms.

## 1. Introduction

Microorganisms residing in the oral cavity are significant components in altering the balance between health and sickness, and include several hundred to thousands of diverse species. The mouth contains bacteria, archaea, protozoa, fungi, and viruses, although each has its own unique characteristic “fingerprint,” usually living in symbiotic harmony with the host (Palmer, 2014; Nobbs and Jenkinson, 2015; Nearing et al., 2020). In the last decade, Human oral microbiome database (HOMD) has been created to provide the scientific community with comprehensive curated information on the bacterial species inhabited in the human aerodigestive tract. Potentially, 774 oral bacterial species exist, approximately 58% of which are officially named, 16% are unnamed but can be cultivated, and the remaining 26% are known only as uncultivated phylotypes (Human Oral microbiome Database. Available at http://www.homd.org), which is currently identified with advances in microscopy and other approaches. Therefore, our understanding of microorganisms has become increasingly profound. An imbalance in a complex ecological community may affect oral and systemic diseases (Abusleme et al., 2013; Hong et al., 2015; Gomes-Filho et al., 2016; Slocum et al., 2016; Xiao et al., 2017; Duncan et al., 2019; Hu et al., 2019; Li et al., 2019; Yu et al., 2020; Corralo et al., 2021). Distinguishing regularly colonizing microorganisms from harmful ones is an important task in this field. Even detecting harmful microorganisms does not necessarily lead to disease. Linking the rapid detection of harmful oral microorganisms with disease diagnosis and precise treatment is the main focus of our efforts. In this review, we focused on the microbial variety in the mouth, presented the microbial community of diverse oral niches, and confirmed the relationship between dysbiosis and oral or systemic disorders.

## 2. Characteristics of oral microbiota

### 2.1. Composition

#### 2.1.1. Bacteria

Bacteria comprise the majority of oral organisms. In 1881, Robert Koch developed a solidified culture medium and a reproducible technology for growing and isolating pure cultures of microbiota with gelatin and later agar, which advanced the identification, analysis, and classification of different microbes (Brock, 1961). In addition, these uncultivable phylotypes are widely observed through molecular taxonomy, particularly 16 s rRNA profiling, next-generation sequencing (NGS) technology, DNA microarrays, and metagenome sequencing (Willse et al., 2005; Claesson et al., 2010; Ghurye et al., 2016; Yang et al., 2016; Vincent et al., 2017; Miao et al., 2018; Gu et al., 2019). The HOMD includes 789 taxa in 19 phyla, including *Bacteroidetes*, *Chlamydiae*, *Chloroflexi*, *Chlorobi*, *Actinobacteria*, *Firmicutes*, *Fusobacteria*, *Gracilibacteria (GN02)*, *Saccharibacteria (TM7)*, *Spirochaetes*, *WPS-2*, *Proteobacteria*, *Euryarchaeota*, *Synergistetes*, *SR1*, *Cyanobacteria*, *Ignavibacteriae*, *Lentisphaerae*, and *Tenericutes* (HOMD).^1^

#### 2.1.2. Fungi

In recent reviews, the oral presence of fungi is purportedly associated with pathologies, since its colonization is presented as a cause of disorders (Jenkinson, 2011; Wright et al., 2013; Chevalier et al., 2018; Anuta et al., 2022; Cannon, 2022). However, an alternative role for fungi in good oral health was reported by Ghannoum et al. (2010). They revealed the “basal” oral microbiota profile in 20 healthy individuals, and demonstrated that a total number of 101 fungal species are identified. It has also been reported that the number of species present in each participant ranged from 9 to 23. And *Candida* species were the most frequently obtained (isolated from 75% of subjects), followed by *Aureobasidium*, *Cladosporium*, *Cryptococcus*, *Saccharomycetals*, *Aspergillus*, *Fusarium*. Four of these predominant genera are pathogenic in humans (Ghannoum et al., 2010). In another study, at least 81 genera and 154 fungal species were observed in 30 adult individuals (15 with oral health and 15 with periodontitis), and *Candida* and *Aspergillus* were the most abundant genera, followed by *Penicillium*, *Schizophyllum*, *Rhodotorula*, and *Gibberella*. *Aspergillus niger* was the most frequently identified species in the samples (Peters et al., 2017).

#### 2.1.3. Viruses

Most viruses present in the mouth are associated with various diseases. Human papilloma virus (HPV) infection can cause many oral lesions, the well-known benign clinical presentations are oral papilloma, condyloma, and focal epithelial hyperplasia (Jiang and Dong, 2017; Syrjanen, 2018; Moran-Torres et al., 2021; Santacroce et al., 2021). Persistent HPV infection is mandatory in HPV-induced malignancies. Herpes simplex virus contributes to herpetic gingivostomatitis, herpes labialis, herpes genitalis, herpetic paterecleris, and mucocutaneous orofacial disease (Dai and Zhou, 2018; Crimi et al., 2019; Atyeo et al., 2021). HIV-related lesions include oral hairy leukoplakia, oral candidiasis, linear gingival erythema, necrotizing ulcerative periodontitis, Kaposi sarcoma, and non-Hodgkin lymphoma (Moyes et al., 2016; Ottria et al., 2018; Polvora et al., 2018; Santella et al., 2019; Sharma Mahendra et al., 2020; Perez Rosero et al., 2021).

#### 2.1.4. Protozoa

Compared with other microbial groups, protozoa constitute a small fraction. The first studied and the most parasite in the oral cavity is *Entamoeba gingivalis*, followed by *Trichomona tenax* (Abualqomsaan et al., 2010; Ghabanchi et al., 2010; Rashidi Maybodi et al., 2016; Yazar et al., 2016; Fadhil Ali Malaa et al., 2022). These protozoa are mainly non-pathogenic commensals under normal conditions. This is characteristic of patients with poor oral hygiene and partial or total edentulism. A Jordanian study designed to evaluate the prevalence of *E*. *gingivalis* and *T*. *tenax* in the oral cavity of 53 patients with gingivitis, 90 patients with periodontitis, and 94 healthy subjects found 88.9% periodontitis patients infected with *E*. *gingivalis*, 84.9% among patients with gingivitis, and 47.9% in healthy controls. For *T*. *tenax*, the percentage was 25.6% among periodontitis patients, 5.7% among gingivitis participants, and 3.2% in the control group. Colonization by *E*. *gingivalis* and *T*. *tenax* was significantly correlated with periodontal disease compared to the healthy cohort (Yaseen et al., 2021).

#### 2.2. Colonization

Recent estimates reveal that the microbial community or biofilm is functionally and structurally organized and attached to surfaces such as the cheek, tongue, hard and soft palate, teeth, and gingival tissue (Zaura et al., 2009; Arweiler and Netuschil, 2016). The microbiota that reside and prevail on particular surfaces depends on the anatomic and biological features of each site (Mager et al., 2003, 2005). As they are easily sampled and accessible, microbiology can be the most studied.

#### 2.2.1. Saliva

Saliva represents the “planktonic phase” of microorganisms. Each milliliter of saliva contained up to 10^9^ microbiota, similar to the bacterial laboratory fluid cultures. Since saliva is swallowed continually, 5 g of bacteria probably flows into the stomach daily. As a result, saliva is not assumed to contain its own inhabitant microbiota. Although most populations share similar salivary microorganisms, differences in salivary composition exist between individuals. Moreover, the microbial composition did not exhibit geographical distribution features. Salivary organisms may be indicators of disease diagnosis. For instance, patients with dental caries, pulpitis, chronic periodontitis, and oral potentially malignant disorders have a distinctive salivary bacterial composition or distribution in healthy individuals (Mager et al., 2005; Belstrom et al., 2014, 2015; Gao et al., 2016; Wang X. et al., 2017; Khurshid et al., 2018).

#### 2.2.2. Surfaces of hard tissues

Non-shedding surfaces of natural teeth or artificial material surfaces of dentures, oral implants, orthodontic appliances, and tooth fillings may be a stable location for the formation of dental plaque, which is a structurally and functionally organized microbial biofilm (Marsh, 2006; Costalonga and Herzberg, 2014). Biofilm formation is divided into supragingival and subgingival biofilms according to the location.

Supragingival plaque (above the gum line) contained a mixture of facultative and anaerobic species. In a Turkish study involving 26 caries-active patients and 26 caries-free controls, testing for dental supragingival plaque, the bacteria responsible for caries was defined as *Anaeroglobus*, *Atopobium*, *Bifidobacterium*, *Centipeda*, *Cryptobacterium*, *Desulfobulbus*, *Filifactor*, *Howardella*, *Lactobacillus*, *Leptotrichiaceae* (unclassified), *Megasphaera*, *Mycoplasma*, *Olsenella*, *Phocaeicola*, *Propionibacterium*, *Pseudoramibacter*, *Scardovia*, *Schwartzia*, *Treponema*, *and Veillonellaceae* (unclassified; Celik et al., 2021). Another study identified 13 genera that were highly abundant in supragingival plaque samples, including *Corynebacterium*, *Capnocytophaga*, *Fusobacterium*, *Leptotrichia*, *Actinomyces*, *Streptococcus*, *Neisseria*, *Haemophilus/Aggr*, *Porphyromonas*, *Rothia*, *Lautropia*, *Veillonella*, *Prevotella* (Mark Welch et al., 2016).

Subgingival plaque (below the gum line) contains more anaerobic genera owing to extreme circumstances, causing periodontitis. Griffen et al. (2012) identified 123 species at drastically higher levels in subjects with chronic periodontitis and 53 species in healthy individuals. *Spirochaetes*, *Synergistetes*, and *Bacteroidetes* were more abundant in the disease group, whereas *Proteobacteria* were significantly more abundant in healthy controls. Within the phylum, *Firmicutes*, *Clostridia*, *Negativicutes*, and *Erysipelotrichia* were correlated with disease, while the class *Bacilli* was health-associated (Griffen et al., 2012). Another study characterizing the subgingival microbiota of 22 patients with periodontal disease and 10 healthy individuals suggested that periodontitis communities had higher percentages of *Spirochetes*, *Synergistetes*, *Firmicutes*, and *Chloroflexi*, among other taxa, whereas the percentages of *Actinobacteria*, particularly *Actinomyces*, were higher in healthy individuals (Abusleme et al., 2013).

#### 2.2.3. Surfaces of soft tissues

**Figure 1:**
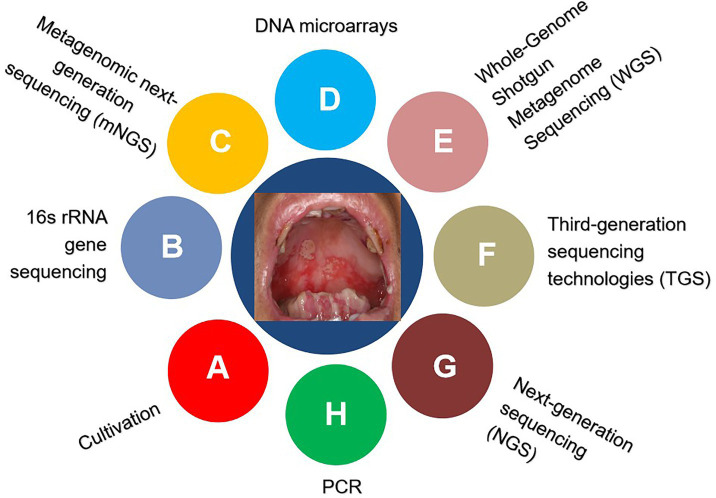
Detection techniques for identifying microorganisms.

**Figure 2:**
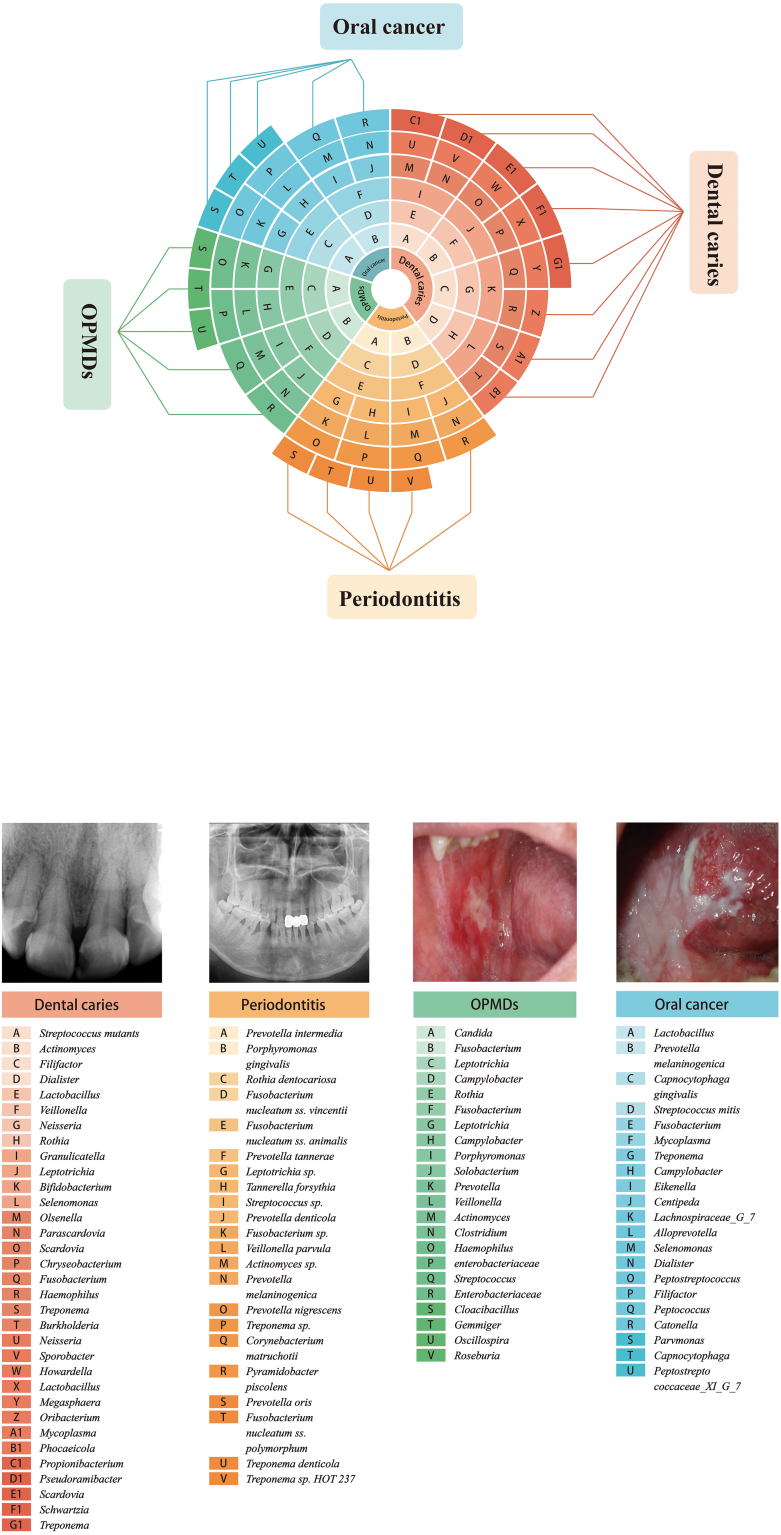
Oral species associated with major oral diseases.

The colonization of microbiota on shedding surfaces, such as lips, cheeks, and palates, is limited owing to bacterial monolayers (He et al., 2015). In contrast, the papillary surface of the tongue has multiple layers of microbiota because of the safe environment provided by the papillae. In addition, tongue microbiota populations are closely related to oral malodor (Tanaka et al., 2004). A study designed by Kazor et al. (2003) to analyze the bacterial diversity on the surface of the tongue dorsum showed that the species predominant in healthy individuals were *Streptococcus salivarius*, *Rothia mucilaginosa*, and an uncharacterized species of *Eubacterium* (strain FTB41; Kazor et al., 2003). Although a large number of bacteria may reside on the tongue, epithelial desquamation restricts microbial colonization on mucosal surfaces. The epithelial shedding squamae was ingested with inhabitant microbes. Therefore, residents need to read the newly developed epithelium (Figures 1, 2).

### 2.3. Mutual translocation

While some species prefer specific habitats, others are found in multiple microniches within the oral cavity. According to physical and morphological criteria, the oral cavity can be divided into several major ecological communities, including the palate, dorsum of the tongue, floor of the mouth, buccal epithelium, supragingival plaque, and periodontal pocket. For example, *P*. *gingivalis*, *P*. *intermedia*, *A*. *actinomycetemcomitans*, *Spirochetes* are able to locate all of these areas (Marsh et al., 2009). A specific example revealed *P*. *melaninogenica* not only in pockets and supragingival plaques, but also in the saliva, tonsils, and other mucosal sites (Quirynen et al., 2001). The findings show that intraoral translocation of periodontopathogens inhibits guided tissue regeneration and locally applied antibiotics, thus jeopardizing the outcome of periodontal therapy (Quirynen et al., 2001).

## 3. An overview of experimental approaches to study oral microbiota

Historically, the cultivation of oral bacterial species requires distinctive conditions, such as biochemically defined media, an anaerobic environment, proper incubation temperature, and diverse PH (Brock, 1961; Kilian, 2007; Loeffelholz and Sanden, 2007; Marmonier, 2007; Vartoukian et al., 2010). However, not all bacteria can be cultivated in the laboratory (Black, 1886; Wade, 2002, 2013; Sizova et al., 2012; Leys et al., 2013; Siqueira and Rocas, 2013). The currently developed culture-independent approaches enable us to identify, classify, and characterize the uncultivable microbiological genera, as summarized in Table 1 and Figure 1 (Liu et al., 2012; Lagier et al., 2015; Krishnan et al., 2017; Benn et al., 2018; Verma et al., 2018).

**Table 1:** Various techniques used for identifying oral microbes.

Techniques	Principles	Applications	Advantages	Limitations
Cultivation	Microorganisms can be grow in certain conditions such as suitable media, temperature and PH et al	Identification of alive microbes; antibiotic susceptibility testing	Economic, effective, gold standard for microbial identification	Laborious and time-consuming; not all organisms can be cultivated
PCR	*In vitro* nucleic acid replication up to millions	Comprehensively used in diagnostic microbiology	High sensitivity /specificity, simplicity of use, and rapid turnaround time, gold standard for molecular detection	Low throughput; not suitable for discovering novel microbes; cannot differentiate dead from living organisms
16 s rRNA gene sequencing	16 s rRNA gene serves as an evolutionary clock and taxonomic signature of bacteria	Extensively used to disclose the microbial diversity and phylogenetic analysis	Culture-independent and sequencing-based approaches for bacterial identification and taxonomic analysis	Limited to bacteria and archaea, while viruses and fungi are missed; genus-level determination of bacteria
DNA microarrays	Hybridization of labeled DNA fragments to large scale of complementary probes fixed onto glass slides, microspheres, or beads	Pathogens identification	Simultaneously detect thousands of pathogens including bacteria, viruses, fungi, and protozoa in a single assay; High throughput and fast	Only targeting the known microbes; lower sensitivity than PCR
Next-generation sequencing (NGS)	Solid-phase bridge amplification or emulsion PCR followed by sequencing during synthesis	Widely used to study microbiota	Simultaneously and independently deep sequencing millions of nucleic acid fragments	In terms of read length and accuracy, NGS technologies are inferior to Sanger sequencing
Metagenomic next-generation sequencing (mNGS)	Deep sequencing the entire set of nucleic acids from a given sample	Assessing which organisms are present and in what proportions in a sample	Unbiased hypothesis-free detection; covering cover both DNA and RNA derived from bacteria, viruses, fungi, and parasites; uncovering novel micororganisms; also offering metatranscriptomic data	Potential cross-contamination; Clinical interpretation of mNGS reports, distinguishing carrier state/colonization and infection, remains challenging; Lack of standardization; mNGS does not detect all pathogens equally
Whole-genome sequencing (WGS)	Amplification of randomly sheared DNA fragments and sequencing the entire genomes	Characterization of the complete genomes	Full genome analysis; offering species-or even strain-level taxonomic resolution	Costly and time-consuming; high-complexity technology
Third-generation sequencing (TGS)	The Nanopore sequencing is mediated by the translation of an electrical signal into a sequence of nucleotides; the PacBio sequencing uses single molecule real-time sequencing technology (SMRT) to sequence the DNA molecule based on a zero-mode waveguide (ZMW) nanostructure	Single-molecule sequencing used for deciphering the complex microbial ecosystems	Long-read sequencing; higher resolution of nucleotide sequences; without PCR amplification; capable of directly identifying native base modifications and sequencing viral RNA genomes in native status	Higher error rate compared to NGS

### 3.1. Cultivation

A pure microbial culture is essential for research on its pathogenesis, antibiotic resistance, and invasive potential, to improve the knowledge and therapy of related infectious diseases. Overall, there are four primary elements involved in the growth of bacteria: nutrients, temperature, atmosphere, and incubation time. However, culture should be more pertinently accommodated for particular species in some cases to provide an optimal environment for their growth. Despite prominent advances in bacterial culture over the past decades, several unsatisfactory results remain. On the one hand, there are some limitations to displaying microbial diversity. However, a large number of organisms may not be cultivated by the conventional method because of their association with other microbiota. Instead of isolation, oral bacteria survive and develop in a complicated community called biofilms (Jenkinson, 2011; Soro et al., 2014). To date, almost 50% of oral microbial species detected through culture-independent gene sequencing techniques remain resistant to cultivation (Sizova et al., 2012; Siqueira and Rocas, 2013; Wade, 2013).

### 3.2. Polymerase chain reaction

Polymerase chain reaction is a revolutionary *in vitro* replication technique that enables the amplification of targeted genes up to millions in a 2–3 h timeframe. Owing to its high sensitivity/specificity, simplicity of use, and rapid turnaround time, PCR has been comprehensively used in diagnostic microbiology. In recent decades, multiple PCR variations have been developed for various purposes, including nested PCR, asymmetric PCR, qualitative PCR, and reverse transcription-PCR (Sakamoto et al., 2005). For example, Deng et al. (2012) established a novel strategy for rapid colorimetric analysis of *Bacillus anthracis* by combining asymmetric PCR with gold nanoparticles. To improve the positivity rate of skeletal tuberculosis diagnosis, which is usually performed by histopathology, researchers have investigated the clinical utility of qPCR-based diagnosis of skeletal tuberculosis using formalin-fixed paraffin-embedded tissues. Compared to that of traditional acid-fast bacillus staining, qPCR offers superior accuracy for mycobacterial detection in clinical samples (He et al., 2022). With the advent of the 2019 coronavirus disease (COVID-19) pandemic, RT-PCR is currently considered the gold standard for COVID-19 detection (El-Kafrawy et al., 2021). Importantly, real-time PCR/RT-PCR can determine experimental results without post-PCR procedures, such as gel electrophoresis, minimizing potential cross-contamination. Notably, molecular methods cannot be used to differentiate between dead and living organisms. In addition, PCR is not well suited for identifying novel microbes. In addition, it only detects a single or small number of gene regions/loci of interest at a time, as with multiplex PCR (Lochman et al., 2019). Therefore, high-throughput approaches are required.

### 3.3. 16S rRNA gene sequencing

As discussed above, culture-dependent methods and PCR-based techniques have limitations in the characterization of bacterial diversity. Owing to developments in sequencing-based approaches, such as 16 s rRNA gene sequencing, this weakness can now be overcome. The 16S rRNA gene is one of the most commonly analyzed marker genes because its functional consistency of 16S rRNA enables it to serve as an evolutionary clock and taxonomic signature in bacterial systematics (Woo et al., 2008). 16S rRNA gene sequencing has been extensively used to determine microbial diversity and for phylogenetic analyses (Janda and Abbott, 2007). PCR-amplified 16S fragments were cloned, sequenced, and clustered based on sequence similarity to infer likely taxonomy. Olson et al. (2011) identified different bacterial signatures in the subgingival plaque of subjects with low or high oral disease by broad-range PCR amplification of the 16S rRNA gene with universal bacterial primers followed by clone-by-clone sequencing. They found that high oral disease exhibited markedly increased bacterial diversity and included an elevated frequency of Clostridiales cluster bacteria, indicating that this atypical bacterial signature is associated with high oral disease (Olson et al., 2011). Another study revealed that anaerobes and oral bacteria are more frequently identified in patients with community-acquired pneumonia than previously thought. These findings strongly suggest that these bacteria may play important in pneumonia (Yamasaki et al., 2013).

Notably, the use of 16S rRNA gene sequencing for bacterial identification can usually be determined accurately at the genus level and not at the species level (Kilian et al., 2016). In addition, this technique is limited to bacteria and archaea, but cannot detect viruses and fungi. Nuclear ribosomal Internal Transcribed Spacer (ITS) regions, including ITS1 and ITS2, are relatively conserved among fungi. Therefore, DNA metabarcoding markers can be used to characterize the diversity and composition of fungal communities (Mbareche et al., 2020).

### 3.4. DNA microarray

In contrast to low-throughput molecular techniques, which generally discover a limited number of targets simultaneously, DNA microarrays offer the benefits of broad coverage, speed, and moderate cost. In recent decades, microarrays have become a viable alternative, enabling simultaneous detection and quantification of hundreds or thousands of targets in a single assay. As a three-dimensional array, suspension microarrays have faster hybridization kinetics and more flexibility in array construction than solid-phase arrays. Briefly, the procedure consists of broad-range PCR, probe design, probe immobilization to microspheres/beads, and molecular hybridization.

Wang et al. (2002) constructed a DNA microarray system, designated ViroChip, for virus identification. Later, GreeneChip and MDA microarrays were developed to detect several thousand pathogens, including viruses, bacteria, fungi, and protozoa (Palacios et al., 2007; Gardner et al., 2010). Common features of these platforms are the use of long (60-mer) oligonucleotide probes and random amplification of nucleic acids. Recently, Huang et al. (2013) developed a high-density microarray platform for large-scale vertebrate pathogen discovery based on similar strategies. This EOPM Chip can detect and distinguish all 2,554 known vertebrate virus species, 124 bacterial genera, 38 fungal genera, and 47 parasitic genera at the species or genus level, nearly all of which are known. Despite its lower sensitivity than that of PCR, this system provides sufficient sensitivity to identify the key pathogens responsible for the clinical symptoms. The authors successfully identified adenovirus causing an outbreak of flu-like infections, as well as cardiovirus in a hand-foot-and-mouth juvenile patient in clinical samples, demonstrating its potential for clinical applications. Theoretically, this cutting-edge platform can be applied for the molecular detection of oral microbes.

The Human Oral Microbe Identification Microarray (HOMIM), a 16S rRNA-based mid-density array containing more than 400 probes, was developed and utilized to analyze oral bacteria. To improve our in-depth understanding of the relationship between the oral microbiota and human health, oral diseases such as periodontitis were investigated using the HOMIM technology (Lourenco et al., 2014). HOMIM analysis of salivary microbiota revealed that a total of 16 bacterial species showed differences between pancreatic cancer samples and controls (Farrell et al., 2012). Advances in NGS have resulted in the upgrading of the HOMIM platform by HOMINGS,^2^ covering approximately 600 oral bacterial taxa to date.

More recently, a new phylogenetic DNA microarray (OralArray) containing 22 probe sets targeting bacteria was designed for the systematic analysis of bacterial communities in different microbial ecosystems, including the oral microbiota (Parolin et al., 2017). Unlike other DNA microarrays, whose discriminatory power is based on DNA hybridization, OralArray is based on Ligation Detection Reaction technology associated with Universal Arrays, which possesses excellent discriminative power and good performance in terms of sensitivity. Validation of OralArray on oral samples demonstrated its ability to efficiently detect the most representative bacterial populations colonizing the oral cavity under physiological and pathological conditions.

### 3.5. Next-generation sequencing

Continuous improvements in DNA sequencing technology over the past few decades have dramatically improved molecular diagnostics. In contrast to first-generation sequencing, NGS technologies are characterized by “sequencing during synthesis” and “massive parallel sequencing” with the ability to simultaneously and independently sequence millions of nucleic acid fragments. NGS enables direct and comprehensive investigation of microbial communities in a single test and provides deeper insights into the microorganisms involved in human diseases (Jo et al., 2016). In recent decades, NGS has gained wide usage in many scenarios owing to its high-throughput capacity, fast turnaround time, and high cost-effectiveness. For example, high-throughput sequencing of the V3-V4 hypervariable regions of the 16S rRNA gene on the Illumina Miseq PE300 platform revealed clear oral microbial dysbiosis among individuals with severe dental caries of primary Sjögren’s syndrome (pSS). Moreover, Veillonella has been proposed as a potential biomarker in patients with pSS (Zhou et al., 2018). These findings may facilitate our understanding of the link between microbial communities and oral homeostasis and human diseases. To study and clarify whether saliva represents a suitable sample for monitoring supragingival microbiota in healthy children, the 16S rRNA gene amplicon was subjected to NGS using the Illumina Miseq system. Sequence reads were clustered into operational taxonomic units (OTUs) after mapping to bacterial reference databases and representative OTU sequences were used for taxonomic identification and abundance estimation. Compared with supragingival plaque, saliva had a less even and less diverse community. *Rothia* and *Streptococcus* were found to discriminate between saliva and plaques. In contrast, supragingival microbiota also exhibited positive associations with salivary microbiota (Shi et al., 2018). It would be interesting to examine the impact of different delivery modes on oral microbiota in healthy infants. 16S rRNA gene-based high-throughput sequencing revealed that newborns delivered *via* cesarean section and individuals delivered vaginally had distinct oral bacteria. The dominant bacteria in the cesarean section group included *Petrimonas*, *Bacteroides*, *Desulfovibrio*, *Pseudomonas*, and *Staphylococcus*. The most abundant genera in the vaginal delivery group were *Lactobacillus*, *Prevotella*, and *Gardnerella* spp. Moreover, the results suggest that repeated sterilization of the vulva might influence infants’ oral microbiota, and attention should be paid to clinical practice (Li et al., 2018b). Similar studies using this technique have been reported previously (Ren et al., 2017; Richards et al., 2017).

Because of the exceptional increase in the number of sequence reads and the reasonable cost, NGS has been widely used to assess the diversity and composition of bacterial ecosystems. However, NGS-based 16S rRNA gene sequencing still focuses only on bacteria and does not cover fungi and viruses. NGS technologies are inferior to Sanger sequencing in terms of the read length and accuracy. In addition, chimera generation during PCR and the intrinsic error rate of sequencing are major concerns. Optimization of the algorithms and experimental procedures is required to solve these problems.

### 3.6. Metagenomic next-generation sequencing

Metagenomic NGS is defined as the process of profiling the entire set of nucleic acids from a given sample that may contain completely different kingdoms of microbes. It sequences and assigns millions of reads to corresponding reference genomes for taxonomic analysis. This powerful technique allows for an unbiased approach to assess the presence of microorganisms and their proportions in a sample. Unlike targeted methods (closed-ended analysis), unbiased hypothesis-free detection (open-ended analysis) is a major advantage of NGS. This technique is sometimes called shotgun sequencing; therefore, it can cover both DNA and RNA derived from bacteria, archaea, viruses, fungi, and parasites, making the diagnosis of mixed infections/co-infections possible. mNGS is akin to “searching for a needle in a haystack” screening technique and theoretically it can detect almost any pathogens in a single run that can never be achieved by traditional approaches (Simner et al., 2018; Gu et al., 2019). During the incipient stage of the COVID-19 pandemic, mNGS offers an accurate and rapid diagnosis compared to previous methods (Chen et al., 2020).

Because mNGS transforms our understanding of the microbial makeup of a mixed sample and the correlation between microbial inhabitants and human diseases, it has been extensively applied to various clinical scenarios (Palacios et al., 2008; Li et al., 2018a; Wang et al., 2019, 2020; Qian et al., 2020; Gu et al., 2021). In addition to depicting microbiota DNA sequences, mNGS can also offer metatranscriptomic data, providing higher-dimensional information. Thus, it can address several issues, such as the microbes present, their functions, and how they interact. Undoubtedly, the integration of the metagenome and metatranscriptome will allow researchers to systematically characterize the complex functions of human ecosystems and vastly expand our awareness of microbe-microbe and microbe-host interactions.

Relatively limited data is available regarding its application in oral microbiota analysis (Zhang Y. et al., 2021). Song et al. (2022) used mNGS to investigate potential pathogens in patients with complex oral mucosal infections and oral leukoplakia. mNGS of oral mucosal tissues identified *Candida albicans*, human gamma herpesvirus 4, and many other pathogens within 36 h (Song et al., 2022). These findings demonstrate that mNGS is an efficient and sensitive tool capable of simultaneously detecting almost all bacteria, viruses, and fungi potentially associated with disease progression. In addition, mNGS is less affected by antibiotic use and has a higher positive detection rate than conventional detection methods. Metagenomic analysis of dental swabs and plaques from patients with periodontal disease has revealed a core community of disease-associated microbes. Some functional genes and metabolic pathways involved in glycan biosynthesis and bacterial chemotaxis were over-represented in the periodontal disease microbiota. This work enriches the understanding of the oral microbial community structure and metabolic variation that is closely associated with periodontal health (Wang et al., 2013).

Although mNGS can produce in-depth, unbiased information and uncover novel organisms, some issues and obstacles need to be solved before high-complexity technology can become the mainstream and route approach in real settings (Han et al., 2019). First, given the excellent analytical sensitivity of mNGS, cross-contamination during specimen collection, library preparation, assay runs, and bioinformatics classification is a major concern. Quality controls are needed to check “splashome” and “kitome” contamination batch by batch, otherwise, its specificity remains the proverbial elephant in the room. Second, mNGS did not detect all pathogens equally. For example, mycobacteria may be more difficult to detect because routine lysis and extraction are difficult to release from mycobacteria. Consequently, even a few reads of mycobacteria are often considered positive, and other methods are required for confirmation. The clinical interpretation of mNGS reports, distinguishing carrier state/colonization from infection, remains challenging. In fact, mNGS lacks methodological standardization and consensus criteria for positive/negative results, making the interpretation of the findings somewhat subjective. Bioinformatics analysis is sophisticated, even for professionals. Sometimes, mNGS reports need to be reevaluated. It is essential to highlight that mNGS alone does not establish pathogenicity, and that close cooperation between clinicians and technicians/bioinformaticians is required to discuss the positive, inconclusive, and negative results of mNGS. Currently, no mNGS tests have been approved by the Food and Drug Administration. However, it may be worthwhile because some diagnoses are made solely by mNGS, whereas no other tests provide useful information. Taken together, mNGS has potential as a front-line diagnostic tool in the near future; however, more effort and research are needed to improve its clinical utility.

### 3.7. Whole-genome sequencing

Metagenomics involves WGS where the sequences of entire genomes are randomly sheared and determined by a “shotgun” strategy. WGS can potentially offer species-or even strain-level taxonomic resolution, whereas high-throughput sequencing of the 16S rRNA gene normally profiles the taxonomic composition at the genus or species level in microbiota analyses. Usually, this is preferred term when this technique is used for human whole-genomic analysis.

### 3.8. Third-generation sequencing technologies

Owing to technical restrictions, targeting sub-region sequencing for short fragments represents a historical compromise. For example, most of the 16 s rRNA gene sequencing based on NGS only amplifies a portion of the marker gene, providing relatively limited information for bacterial community characterization. The advent of innovative long-read sequencing technologies, including the PacBio and Oxford nanopore platforms, has changed this and opened new avenues for microbiota studies (Jiao et al., 2013; Li et al., 2016). In addition to maintaining the features of high-throughput and in-depth sequencing, the read lengths are up to 1.0–1.5 × 10^4^ and 2–5 × 10^3^ for PacBio and Oxford nanopore single-molecule sequencing platforms, respectively (Mosher et al., 2014; Laver et al., 2015; van Dijk et al., 2018). However, TGS is capable of routinely generating reads in excess of 1,500 bp and completely covering the full 16S rRNA gene (Franzen et al., 2015; Schloss et al., 2016; Wagner et al., 2016). Previous studies have shown that TGS of the full-length (V1-V9) 16S rRNA gene using the PacBio technique generated sufficient accuracy to resolve subtle nucleotide variations for taxonomic analysis at species or even strain levels (Earl et al., 2018; Johnson et al., 2019). Moreover, TGS has additional advantages over NGS platforms for deciphering complex microbial ecosystems. For example, nanopore sequencing is mediated by the translation of an electrical signal into a sequence of nucleotides and does not involve nucleic acid amplification. In addition, nanopore sequencing can directly identify native base modifications and sequence viral RNA genomes in native status (Quick et al., 2016; Rand et al., 2017; Simpson et al., 2017; Garalde et al., 2018; Liu et al., 2019; Freed et al., 2020). Consequently, this substantially eliminates the biases introduced by PCR or reverse transcription. Many critical human diseases are caused by emerging RNA viruses such as COVID-19, severe acute respiratory syndrome, and Ebola.

To assess the performance of different high-throughput sequencing platforms in analyzing oral microbial communities and their relation to health, saliva samples from five healthy preschool children were collected and subjected to sequencing on the MiSeq (targeting V3-V4) and PacBio RS II (targeting V1-V9) platforms. Although the PacBio system provided a lower amount of clean data, it had a longer read length and higher resolution of nucleotide sequences at the species or strain level than those of NGS (Wang Y. et al., 2017; Zhang et al., 2020). Long-read sequencing of two independent oral *S*. *epidermidis* isolates was conducted using the Oxford Nanopore MinION sequencing platform. TGS results revealed that both isolates harbored a novel structural organization. The identification of independent genetic evolution further illustrates the diversity of arginine catabolic mobile element elements in *S*. *epidermidis*, which may confer a selective advantage to oral *S*. *epidermidis* in dental plaque (McManus et al., 2019).

The main drawback of TGS is its high error rate, which has been significantly improved (Rang et al., 2018). The state-of-the-art methodologies open up more possibility of identifying microorganisms as a second wave of technical advances in the recent decade (Ciuffreda et al., 2021), it should have a wider application in medical practice.

## 4. Microbial dysbiosis and oral pathology

### 4.1. Dental caries

Dental caries, one of the most prevalent oral diseases, usually leads to tooth pain and pulp or periapical infections. This process involves three reversible stages. In the first stage, acidification is mild and infrequent, resulting from a microbial biofilm containing mainly non-mutans *Streptococcus* and *Actinomyces* (Takahashi and Nyvad, 2008). This is consistent with the balance of the demineralization/remineralization equilibrium or the conversion of the mineral balance toward net mineral gain. Once a continuous sugar supply is established, acidification changes moderately and frequently, which may improve the capability of non-mutans bacteria to produce acid and resist PH reduction. Therefore, non-mutans *streptococci* and other aciduric strains may selectively increase (Aas et al., 2005, 2008). These acidogenic and aciduric processes of the microorganism may shift the demineralization/remineralization equilibrium toward net mineral loss, resulting in the progression of dental caries (Becker et al., 2002; Martin et al., 2002; Munson et al., 2004; Mantzourani et al., 2009). Under continuous and prolonged acidic conditions, aciduric bacteria such as *mutans streptococci*, *Lactobacillus*, and *Actinomyces* may become dominant through acid-induced selection (Takahashi and Nyvad, 2011). Environmental acidification is a major factor in phenotypic and genotypic changes in microorganisms during the initiation and development of caries.

### 4.2. Periodontitis

Gingivitis is a reversible inflammation caused by the accumulation of bacterial plaque in the gingival tissue. The microbiota involved are commensal microbiota, such as *Actinomyces* species, *F*. *nucleatum*, *Prevotella intermedia*, *Bacteroides*, *Capnocytophaga*, and *Eikenella* (Huang et al., 2011; Igic et al., 2012).

Gingivitis may develop into periodontitis, resulting from loss of control. Periodontal disease is a chronic irreversible inflammation caused by the destruction of gum tissue, alveolar bone, and tooth loss. *P*. *intermedia*, *F*. *nucleatum*, *Peptostreptococcus micros* and *Prevotella nigrescens*, defined as microbiota of the “orange complex,” as well as the “red complex,” composed of *Treponema denticola* (*T*. *denticola*), *Porphyromonas gingivalis* (*P*. *gingivalis*), and *Tannerella forsythia* (*T*. *forsythia*) are the most commonly associated with periodontitis (Socransky et al., 1998). Although these microbial species are present in low numbers in healthy individuals, they are thought to be responsible for the occurrence and progression of the disease (Kumar et al., 2003). These complex microorganisms fall or disappear (below the detection limit) after efficient treatment.

Moreover, the presence of fungi, protozoa, viruses, and methanogenic archaea is highly correlated with the severity of chronic periodontitis (Horz et al., 2015; Zhu et al., 2015; Lauritano et al., 2016; Jabri et al., 2021).

### 4.3. Oral potentially malignant disorders

Oral potentially malignant disorders (OPMDs) are a series of lesions or conditions that may undergo malignant transformation to oral cancer. These diseases are most commonly represented as clinically white patches (oral lichen planus, leukoplakia), red patches (erythroplakia), or red and white patches (erythroleukoplakia). Although the etiopathogenesis of OPMDs is intricate and unclear, contemporary studies have suggested that microbial dysbiosis influences disease development through molecular mechanisms. Significant evidence supports that Candida colonization is regularly correlated with oral leukoplakia (OL), which is called as “Candida leukoplakia,” with infiltration of hyphae in the superficial epithelium (Abdulrahim et al., 2013; Gupta et al., 2019; Weerasekera et al., 2021). *C*. *albicans* is the most common Candida species associated with leukoplakia, and its genotype A strains are more frequently associated with OL transformation (Alnuaimi et al., 2015). Over the last decade, increased attention has been paid to microbial infections in the pathogenesis of oral lichen planus. Recent evidence indicates that bacteria are abundant throughout the epithelium and lamina propria of OLP tissues and are positively associated with the levels of infiltrated T cells and CD3^+^, CD4^+^, and CD8^+^ cells. Therefore, microorganisms may play a key role in OLP onset (Choi et al., 2016; Bombeccari et al., 2017; Baek and Choi, 2018; Yu et al., 2020). The microbiota varies among different OLP types, with higher levels of bacteria and lower levels of fungi. Higher enrichment of the fungi Candida and Aspergillus was identified in patients with reticular OLP than in healthy controls. In addition, Alternaria and Sclerotiniaceaeunidentified were significantly more abundant in patients with erosive OLP (Li et al., 2019).

### 4.4. Oral cancer

Oral squamous cell carcinoma (OSCC) is the most common malignant neoplasm of the oral cavity, accounting for 90% of head and neck cancers. The 5-year survival rate has remained at 50% within the last few decades due to asymptomatic presentation and poor prognosis. The driving factors vary significantly, including smoking, alcohol intake, and betel nut chewing. Recent studies have suggested that changes in the composition of the oral microbiota may play a role in the induction of oral cancer (Mager et al., 2005; Hooper et al., 2009; Schmidt et al., 2014; Srinivasprasad et al., 2015; Deo and Deshmukh, 2020; Stasiewicz and Karpinski, 2022). *P. gingivalis*, a critical pathogen in periodontal disease, is a significant trigger agent for oral cancer, and its colonization in tumor tissues is correlated with poor survival in patients with carcinoma (Karmakar et al., 2020; Nwizu et al., 2020; Kavarthapu and Gurumoorthy, 2021; Soder et al., 2021). A study has shown that the microbial communities in OSCC patients at stage 4 became more complicated compared to those in healthy controls. In addition, microbiological populations were significantly altered by the development of cancer from the early stage to the late stage. At the phylum level, the oral samples showed large changes in the abundances of *Actinobacteria*, *Bacteroidetes*, and *Fusobacteria*. The number of *Fusobacteria* increased significantly during cancer progression in the healthy controls. Although the abundance of dominant genera such as *Fusobacteria* increased with cancer development, the abundance of *Haemophilus*, *Streptococcus*, *Actinomyces*, and *Porphyromonas* decreased. *Parvimona micra*, *Haemophilus influenzae*, *Fusobacterium periodonticum*, *Streptococcus constellatus*, and *Filifactor alocis* progressively increased with oral cancer development (Yang et al., 2018). Another study revealed that the microbial composition of five genera, *Enterococcus*, *Parvimonas*, *Peptostreptococcus*, *Bacillus*, and *Slackia*, showed relatively significant differences between epithelial precursor lesions and cancer tissues. This shift in microbiota might serve as a potential biomarker for monitoring the occurrence and prognosis (Lee et al., 2017). The highly correlated organisms are summarized in Table 2 and Figure 2.

**Table 2:** Oral species implicated in oral diseases.

Diseases and microorganisms	Clinical manifestations	Clinical treatments
Dental caries: *Streptococcus mutants*; *Actinomyces*; *Filifactor*; *Dialister*; *Lactobacillus*; *Veillonella*; *Neisseria*; *Rothia*; *Granulicatella*; *Leptotrichia*; *Bifidobacterium*; *Selenomonas*; *Olsenella*; *Parascardovia*; *Scardovia*; *Chryseobacterium*; *Fusobacterium*; *Haemophilus*; *Treponema*; *Burkholderia*; *Neisseria*; *Sporobacter*; *Howardella*; *Lactobacillus*; *Megasphaera*; *Oribacterium*; *Mycoplasma*; *Phocaeicola*; *Propionibacterium*; *Pseudoramibacter*; *Scardovia*; *Schwartzia*; *Treponema*	The physical and chemical processes of demineralization and remineralization occurring on the tooth surface	Chemoprophylactic agents, antimicrobial peptides, vaccines, probiotics, sugar substitutes, and remineralization agents
Periodontitis: *Prevotella intermedia*; *Porphyromonasgingivalis*; *Rothiadentocariosa*; *Fusobacterium nucleatum* ss. *Vincentii; Fusobacterium nucleatum* ss. *Animalis*; *Prevotellatannerae*; *Leptotrichia* sp.; *Tannerella forsythia*; *Streptococcus* sp.; *Prevotelladenticola*; *Fusobacterium* sp.; *Veillonellaparvula*; *Actinomyces* sp.; *Prevotellamelaninogenica*; *Prevotellanigrescens*; *Treponema* sp.; *Corynebacterium matruchotii*; *Pyramidobacterpiscolens*; *Prevotellaoris*; *Fusobacterium nucleatum* ss. *Polymorphum*; *Treponema denticola*; *Treponema* sp. HOT 237	An inflammatory condition that affects the supporting structures of teeth with the microbial communities that inhabit the subgingival environment serving as the inflammatory trigger	Including oral hygiene instruction and other educational interventions to improve patient motivation and adherence; subgingival instrumentation with or without adjunctive therapies; surgical intervention
OPMDs: *Candida*; *Fusobacterium*; *Leptotrichia*; *Campylobacter*; *Rothia*; *Fusobacterium*; *Leptotrichia*; *Campylobacter*; *Porphyromonas*; *Solobacterium*; *Prevotella*; *Veillonella*; *Actinomyces*; *Clostridium*; *Haemophilus*; *Enterobacteriaceae*; *Streptococcus*; *Cloacibacillus*; *Gemmiger*; *Oscillospira*; *Roseburia*	A group of disorders described as genetically mutated oral epithelial cells with or without clinical and histomorphological abnormal appearances that are malignant transformable	Reducing or abandoning tobacco or alcohol use; increasing the intake of fruit and vegetables in the diet; and possibly the use of active agents
Oral cancer: *Lactobacillus*; *Prevotellamelaninogenica*; *Capnocytophagagingivalis*; *Streptococcus mitis*; *Fusobacterium*; *Mycoplasma*; *Treponema*; *Campylobacter*; *Eikenella*; *Centipeda*; Lachnospiraceae_G_7; *Alloprevotella*; *Selenomonas*; *Dialister*; *Peptostreptococcus*; *Filifactor*; *Peptococcus*; *Catonella*; *Parvmonas*; *Capnocytophaga*; Peptostreptococcaceae_XI_G_7	A subset of head and neck cancer which is a type of cancer that occurs in the oral cavity affecting the lips, tongue, gingiva, floor of mouth, palate and other related structures	Surgical treatment or brachytherapy; postoperative radiotherapy or chemoradiotherapy

## 5. Oral microbiota and systemic condition

### 5.1. Atherosclerotic disease

Atherosclerotic disease, which is characterized by the accumulation of lipids and recruitment of T cells into the arterial wall, is considered a chronic inflammatory disorder. Although a number of traditional risk factors, such as diabetes, obesity, high blood pressure, and smoking are widely accepted, oral infection is increasingly manifested in atherosclerotic plaque samples (Fernandes et al., 2014; Aarabi et al., 2015; Slocum et al., 2016; Atarbashi-Moghadam et al., 2018; Herrera et al., 2020). Chhibber-Goel et al. (2016) demonstrated the presence of 23 oral commensal bacteria, either individually or in coexistence, within carotid endarterectomy biopsies from patients undergoing surgical procedures. Of these 23 bacteria, five were unique to coronary plaques, including *P. gingivalis*, *P. endodontalis*, *P. intermedia*, *C. rectus*, and *P. nigrescens*. The remaining 18 genera, otherwise confirmed in non-cardiac organs, are responsible for over 30 non-cardiac lesions (Chhibber-Goel et al., 2016).

### 5.2. Adverse pregnancy outcomes

Adverse pregnancy outcomes are a broad term containing preeclampsia, low birth weight, preterm premature rupture of membranes, early-onset neonatal sepsis, miscarriage, stillbirth, and fetal growth retardation (Figuero et al., 2020). The relationship between specific pathogenic bacteria and APO has been known for a long time (Komine-Aizawa et al., 2019; Bobetsis et al., 2020; Xu and Han, 2022). The prevalent oral species are *Filifactor alocis*, *Fusobacterium nucleatum*, *Porphyromonas gingivalis*, *Campylobacter rectus*, *T*. *Denticola*, and *T*. *Forsythia*, among others (Cobb et al., 2017). Stimulated fetal inflammatory and immune responses may ultimately increase the potential for APO owing to the virulent properties assigned to microbiota in the intrauterine environment.

### 5.3. Diabetes mellitus

Diabetes mellitus is a metabolic syndrome characterized by hyperglycemia caused by a defect in insulin secretion, decreased insulin action or both (Harreiter and Roden, 2019). Multiple studies have verified a bidirectional relationship between diabetes and periodontitis. Diabetes leads to a shift in connective tissue metabolism and consequently reduces the capacity of inflammation resolution and remodeling, which results in aggravated periodontal impairment. However, the periodontal condition in the host may negatively affect glycemic control in diabetic subjects and exacerbate diabetes progression and its complications (Lalla and Papapanou, 2011; Preshaw et al., 2012; Bascones-Martinez et al., 2015; Nascimento et al., 2018; Sanz et al., 2018; Baeza et al., 2020; Zhang X. et al., 2021). Casarin et al. (2013) reported dramatic differences in the subgingival ecosystem between individuals with and without diabetes. Compared to the levels in non-diabetic individuals, diabetes patients exhibited a higher abundance of *Neisseria*, *TM7*, *Gemella*, *Eikenella*, *Selenomonas*, *Actinomyces*, *Capnocytophaga*, *Fusobacterium*, *Aggregatibacter*, *Veillonella*, and *Streptococcus* genera and a lower abundance of *Filifactor*, *Synergistetes*, *Tannerella*, *Eubacterium*, *Porphyromonas*, and *Treponema genera* (Casarin et al., 2013).

## 6. Conclusion

The use of the current molecular-based detection approaches has dramatically expanded our awareness of the diversity, architecture, and function of oral microorganisms in health and diseases. Disease conditions supervene when the balance of the microbiota community is disrupted. However, the underlying mechanisms remain unclear. Further research is necessary to accurately identify the pathogenic microbiota. Numerous strategies for defining oral microbes have been investigated and developed. However, future studies must explore the security and efficacy of these methods.

## Author contributions

XX, SL, and HD wrote the manuscript. YS prepared the figures. LZ and ZS reviewed the manuscript. All authors contributed to the article and approved the submitted version.

## Funding

This work was supported by the Natural Science Foundation of Shanghai (No. 22ZR1454200) and Shanghai Stomatological Hospital Talent Project (No. SSDC-2019-RC01) to SL.

## Conflict of interest

The authors declare that the research was conducted in the absence of any commercial or financial relationships that could be construed as a potential conflict of interest.

## Publisher’s note

All claims expressed in this article are solely those of the authors and do not necessarily represent those of their affiliated organizations, or those of the publisher, the editors and the reviewers. Any product that may be evaluated in this article, or claim that may be made by its manufacturer, is not guaranteed or endorsed by the publisher.

## Footnotes

^1^ www.homd.org

^2^ https://microbiome.forsyth.org/
